# Associative effects between *Chlorella vulgaris* microalgae and *Moringa oleifera* leaf silage used at different levels decreased in vitro ruminal greenhouse gas production and altered ruminal fermentation

**DOI:** 10.1007/s11356-022-22559-y

**Published:** 2022-08-20

**Authors:** Ahmed Eid Kholif, Gouda Abdelhaleam Gouda, Tarek Abdelfattah Morsy, Osama Hefiny Matloup, Sobhy Mohamed Sallam, Amlan Kumar Patra

**Affiliations:** 1 Dairy Science Department, National Research Centre, 33 Bohouth St. Dokki, Giza, Egypt; 2 Department of Animal and Fish Production, Faculty of Agriculture, El-Shatby, Alexandria University, Alexandria, Egypt; 3 Department of Animal Nutrition, West Bengal University of Animal and Fishery Sciences, 37 K.B. Sarani, Kolkata, India

**Keywords:** Associative effect, *Chlorella vulgaris*, Methane, *Moringa oleifera* silage, Ruminal fermentation

## Abstract

*Moringa oleifera* leaf silage and *Chlorella vulgaris* microalgae mixture used at different levels replacing concentrate feed mixture in the diets of ruminant were evaluated using an in vitro gas production technique. *C. vulgaris* was included in rations at 1, 2, and 3% concentrations. The concentrate feed mixture was replaced by *M. oleifera* silage up to 100%. Productions of total gas, methane (CH_4_), and carbon dioxide (CO_2_) and ruminal fermentation were measured. Interactions between *M. oleifera* and *C. vulgaris* levels were observed for the rate of total gas production, lag time of CH_4_ production, pH, and concentrations of ammonia-N (NH_3_-N), total volatile fatty acid (VFA), and propionate. The lower level of *C. vulgaris* increased total gas production and decreased CH_4_ and CO_2_ production as well as improved nutrient degradability compared to the other levels of *C. vulgaris* which showed less improvement in these parameters. The replacement levels of concentrate at 10 to 40% with *M. oleifera* linearly increased the asymptotic total gas production and degradabilities of dry matter and acid detergent fiber (*P*<0.05), while the replacement levels of 80 to 100% lowered the asymptotic (*P*<0.01) for the ration containing 1% *C. vulgaris*. Rations containing *M. oleifera* linearly increased the lag time of total gas production (*P*<0.05), neutral detergent fiber degradability, and ruminal bacteria count and decreased the asymptotic CH_4_ and CO_2_ production and ruminal protozoal count (*P*<0.05). For the rations containing 2 and 3% *C. vulgaris*, *M. oleifera* linearly (*P*<0.01) decreased the asymptotic total gas, CH_4_ and CO_2_ production, and ruminal protozoal count. The lag time of CH_4_ production was not affected at 1% *C. vulgaris*, but reduced linearly at 2% and 3% *C. vulgaris*. Ruminal pH was not affected by *M. oleifera*, but was increased by *C. vulgaris* at 3% level. Overall, *M. oleifera* in the ration containing *C. vulgaris* at all levels increased ruminal NH_3_-N concentration; however, *C. vulgaris* at 2% level and *M. oleifera* at levels up to 40% lowered NH_3_-N concentration. *M. oleifera* rations with 1% and 2% *C. vulgaris* increased the concentrations of total VFA and propionate, whereas these variables were not affected at 3% *C. vulgaris* level. In conclusion, replacement of concentrate mixture with *M. oleifera* at 30% level and *C. vulgaris* at 1% in the diet due to associative effects may improve ruminal fermentation and feed degradability while decreasing CH_4_ production.

## Introduction

The Food and Agriculture Organization considers ruminants as one of the main producers of greenhouse gases. Ruminal fermentation of feeds produces about 40% of total anthropogenic emissions of greenhouse gases from livestock systems, resulting in losing energy from digested feeds (Grossi et al. 2019). Thus, reducing ruminal methane (CH_4_) production could improve energy utilization efficiency and reduce environmental burdens within the livestock production industry, which is attributed to the direct reduction of ruminal methanogenesis. Several experiments have proved the ability of secondary metabolites in some plant species to decrease CH_4_ production, improve animal performance, and reduce protein degradation in the rumen (Akanmu and Hassen 2018; Kholif and Olafadehan 2021a; Morsy et al. 2022).

The scarcity and high cost of concentrates when available are one of the main challenges for successful livestock farming. This situation forces animal nutritionists to explore less expensive alternative feeds. Tree leaves and protein-rich microalgae are among the alternative feeds that have gained increasing interest in recent years. *Moringa oleifera* (moringa or drumstick) is a rapid-growing softwood tree that grows in all tropical and subtropical areas with round the year availability. The proximate analysis revealed that *M. oleifera* leaves contain moderate levels of crude protein (CP; 23 to 30%) and fat (4.03 to 9.51%) mainly α-linolenic acid, low levels of crude fiber (6.0 to 20.4%), and high levels of ash (8.1 to 10.4%) including calcium (1.32 to 2.65%) for ruminants. Also, *M. oleifera* leaves contain vitamin C, phosphorus, and potassium (Azzaz et al. 2016; Sultana 2020). The CP in *M. oleifera* leaves has about 47% rumen bypass protein (Su and Chen 2020) and a good amino acid profile (Sánchez-Machado et al. 2010). Additionally, it contains substantial concentrations of several important bioactive compounds including polyphenols (0.21 to 1.22% as gallic acid equivalents), tannins (1.32 to 2.06%), saponins (0.64 to0.81%), carotenoids (0.066 to 0.068% as beta-carotene), antioxidants (up to 8%), and health-promoting phytochemicals including glucosinolates (up to 11.6%) and isothiocyanates (up to 6.3%) (Nouman et al. 2016; Premi and Sharma 2017). However, it contains some antinutritional factors (e.g., oxalates at 4.1% and phytates at 3.1%) (Gupta et al. 1989). Recently, Abdel-Raheem and Hassan (2021) replaced soybean meal in the concentrate mixture with *M. oleifera* leaf meal at 50 and 75% (equal to 15 and 20% of total diet) in the diet of buffalo calves and observed increased feed intake; digestibility of dry matter (DM) and crude fiber; concentrations of ruminal acetic, propionic, butyric acids, and total volatile fatty acid; and improved final body weight and daily weight gain, while decreased the digestibility of CP, activity of ruminal enzymes, concentrations of ammonia-N (NH_3_-N), and total protozoal count. Feeding *M. oleifera* leaves alters ruminal fermentation and inhibits methanogenesis due to their contents of some active compounds such as saponins, tannins, and phenolics (Dong et al. 2019). In an in vitro experiment, Seradj et al. (2019) observed that replacing alfalfa with *M. oleifera* decreased the lag time of gas production and increased organic matter (OM) degradability.

*C. vulgaris* is a fresh-water, unicellular microalgae, which contains high CP (58%) with almost all the essential amino acids (Kholif and Olafadehan 2021b). *C. vulgaris* contains relatively high concentrations of lysine and methionine, which are the first two limiting amino acids in animal nutrition (Kholif et al. 2017). Additionally, *C. vulgaris* contains antioxidants, provitamins, vitamins, pigments, and a growth substance known as the *C. vulgaris* growth factor (CGF), which can stimulate immune responses modulating cytokine production, and enhance feed intake and utilization (Kotrbáček et al. 2015; Ru et al. 2020). Some experiments (Tsiplakou et al. 2017; Kholif et al. 2017) showed improved ruminal fermentation and decreased in vitro CH_4_ production with *C. vulgaris* supplementation. However, other researchers reported that effect of *C. vulgaris* on CH_4_ production is not consistent (Sucu 2020) and is highly influenced by diet (Meehan et al. 2021). Feeding high levels of *C. vulgaris* to ruminants reduced nutrient digestibility due to its rigid cellulosic cell wall structures (Kotrbáček et al. 2015). Individually, *M. oleifera* up to 17.5% of total diet DM (Morsy et al. 2022) or *C. vulgaris* up to 2% (Tsiplakou et al. 2017; Kholif et al. 2017) were reported to improve ruminal fermentation while reducing CH_4_ production.

Greater levels of ingredients containing plant metabolites can impair microbial fermentation and digestibility in the rumen though CH_4_ production could be decreased substantially as noted for some methane mitigating agents (Patra 2016; Kholif and Olafadehan 2021a, b). Some methanogenic agents with complementary modes of action at binary or ternary combinations have been shown to decrease CH_4_ production additively without affecting ruminal fermentation (Patra and Yu 2014). Moreover, the associative effects of two or more protein ingredients in ruminant animals have been proved in many experiments due to the presence of complementary amino acid composition, which supports the idea that the nutritive value of mixing more feeds will improve their nutritive value beyond their individual value (Yuan et al. 2020). Therefore, we hypothesized that combination of both *M. oleifera* silage and *C. vulgaris* at low levels could exert associative effects on ruminal fermentation and feed degradability and additively decrease ruminal CH_4_ production. However, an optimum combination level of these plants is needed to decide for obtaining these responses, if any. Accordingly, this experiment aimed to evaluate different replacement levels of concentrate feed mixture with *M. oleifera* leaf silage in the presence of *C. vulgaris* microalgae on in vitro ruminal production of CH_4_ and carbon dioxide (CO_2_), nutrient degradability, and fermentation profile.

## Materials and methods

### Moringa oleifera cultivation

*M. oleifera* seeds, obtained from The Egyptian Association of Moringa (National Research Centre, Egypt), were planted at a density of 100,000–150,000 seeds per ha. The field was irrigated with 900 m^3^ water/ha biweekly without any fertilizer. When plants reached 65–70 cm height, a first uniformity cutting was carried out at 5–7 cm cutting height 65 days after seeding. This cut was not used in the present experiment. For the in vitro evaluation, a second cut of *M. oleifera* (45 days after the first cut) biomass composed of leaves and small twigs was harvested and large twigs were removed. Usually, *M. oleifera* gives 9 harvests per year and yielding 70–80 tons of fresh biomass/ha/year (∼23 tons DM/ha/year). The material (about 1 ton) was left on the field for 1 h and then chopped and used to prepare silage. Sugarcane molasses was mixed at 5% of fresh weight. The materials (about 40 kg fresh materials per bag) were then packed into a polythene silo bag (40 × 70 cm) and compressed manually for quick creation of semi-anaerobic conditions. The bags (25 bags) were sealed and stored indoors on a dry concrete floor for 45 days. Before using the silage in the in vitro experiment, 5 kg of ensiled materials (collected from 5 different bags; 1 kg/bag) was dried and kept for evaluation and chemical analysis.

### Chlorella vulgaris microalgae cultivation

Laboratory production of *C. vulgaris* was performed using 5-L glass flasks containing 3 L algal growth medium. Pure strain of *C. vulgaris* H1957 was obtained from the Marine Toxins laboratory, National Research Centre, Egypt. The culture medium used for cultivation of *C. vulgaris* was BG-11 medium (Rippka et al. 1979). After autoclaving and cooling, pH of the medium was adjusted to 7.1. *C. vulgaris* was cultivated under continuous illumination coming from white fluorescent lamps at room temperature and aeration was performed using an air compressor linked with polyethylene tubes (3 mm). After 25 days, *C. vulgaris* growth culture in its late exponential phase was transferred at 1:10 into 1000-L polyethylene tanks (*n* = 5) containing 600 L culture media and linked with an aeration system. *C. vulgaris* biomass harvesting was performed using the continuous separating centrifuge apparatus (Westfalia Separator centrifuge at 15,000 L/h) and drained water was recycled to the ponds. The harvested biomass (0.75 kg microalgae per day) was re-washed three times with tap water to remove any residues of salts from the culture media. Biomass was partially dried using an air-drying oven at 45°C for 2 to 4 h.

### Experimental rations

Four rations were formulated to contain (DM basis) (1) 40% berseem hay (*Trifolium alexandrinum*), 10% ensiled vegetable and fruits byproducts (bought from local markets and based mainly on carrot roots, tomatoes, cabbage leaf, and courgette at 1:1:1:1 DM weight; ensiled for 45 days under semi-anaerobic conditions without any additives), and 50% concentrate mixture without *C. vulgaris* microalgae or ensiled *M. oleifera*; (2) 40% berseem hay, 10% ensiled vegetable and fruits byproducts, 49% concentrate mixture, and 1% *C. vulgaris* microalgae; (3) 40% berseem hay, 10% ensiled vegetable and fruits byproducts, 48% concentrate mixture, and 2% *C. vulgaris* microalgae; and (4) 40% berseem hay, 10% ensiled vegetable and fruits byproducts, 47% concentrate mixture, and 3% *C. vulgaris* microalgae. The concentrate mixture contained 25% un-decorticated cotton seed meal, 35% wheat bran, 30% maize, 3% rice bran, 3% molasses, 2% limestone, 1% urea, and 1% salt. In each of the formulated rations containing *C. vulgaris* microalgae (i.e., ration 2, 3, and 4), concentrate feed mixture was replaced with dried *M. oleifera* silage at 10, 20, 30, 40, 50, 60, 70, 80, 90, and 100%. The chemical composition of ingredients (Table 1), proportion of ingredients in formulated ration (Table 2), and chemical composition of the formulated rations used as substrates (Table 3) have been tabulated.

**Table 1.** Chemical composition (% DM), concentrations of phenolic compounds (% DM), and silage quality of *M. oleifera* silage, ensiled vegetable/fruit byproducts, and *C. vulgaris* microalgae

	*M. oleifera* fresh leaves and twigs	*M. oleifera* silage^1^	Microalgae	CFM^1^	Berseem hay	Ensiled vegetable/fruit byproducts
Dry matter	33.3	39.1	93.2	83.8	86.0	22.9
Organic matter	90.1	86.2	94.2	89.1	85.8	94.1
Crude protein	29.3	28.2	57.9	16.2	19.3	5.9
Ether extract	4.6	4.5	13.9	4.2	3.2	6.6
Non-structural carbohydrates	21.1	19.0	10.6	42.1	21.7	48.0
Neutral detergent fiber	35.1	34.5	11.8	26.6	41.6	33.6
Acid detergent fiber	30.5	29.9	4.3	9.9	30.2	29.3
Total phenolic	5.5	4.9	ND	ND	ND	ND
Tannins	2.6	1.9	ND	ND	ND	ND
Silage quality				ND	ND	
pH	ND	4.2	ND	ND	ND	3.7
Ammonia-N	ND	5.1	ND	ND	ND	4.4
Volatile fatty acids	ND	8.8	ND	ND	ND	8.3
Aflatoxin B_1_	ND	0.11	ND	ND	ND	0.4

*CFM*, concentrate feed mixture; *ND*, not determined

^1^Contained: 25% un-decorticated cotton seed meal, 35% wheat bran, 30% maize, 3% rice bran, 3% molasses, 2% limestone, 1% urea, and 1% salt

**Table 2:** Ingredient concentration of rations containing *M. oleifera* silage replacing concentrate mixture at different levels in the presence of three levels of *C. vulgaris* (% DM)

Replacement level^1^	Berseem hay	Ensiled vegetable/fruit byproducts	1% *C. vulgaris*			2% *C. vulgaris*			3% *C. vulgaris*		
			CFM	*M. oleifera* silage	Microalgae	CFM	*M. oleifera* silage	Microalgae	CFM	*M. oleifera* silage	Microalgae
0% (control)^2^	40	10	50.0	0	0	50.0	0	0	50.0	0	0
0%	40	10	49.0	0	1	48.0	0	2	47.0	0	3
10%	40	10	44.1	4.9	1	43.2	4.8	2	42.3	4.7	3
20%	40	10	39.2	9.8	1	38.4	9.6	2	37.6	9.4	3
30%	40	10	34.3	14.7	1	33.6	14.4	2	32.9	14.1	3
40%	40	10	29.4	19.6	1	28.8	19.2	2	28.2	18.8	3
50%	40	10	24.5	24.5	1	24.0	24.0	2	23.5	23.5	3
60%	40	10	19.6	29.4	1	19.2	28.8	2	18.8	28.2	3
70%	40	10	14.7	34.3	1	14.4	33.6	2	14.1	32.9	3
80%	40	10	9.8	39.2	1	9.6	38.4	2	9.4	37.6	3
90%	40	10	4.9	44.1	1	4.8	43.2	2	4.7	42.3	3
100%	40	10	0	49.0	1	0	48.0	2	0	47.0	3

^1^Concentrate feed mixture was replaced by *M. oleifera* silage at different levels (0 to 100%, DM basis) in the presence of three levels of *C. vulgaris* (1, 2, and 3%, DM basis) in the diets

*CFM*, concentrate feed mixture

^2^No *C. vulgaris* or *M. oleifera* silage included in the ration

**Table 3:** Chemical composition (% DM basis, except for DM content) of rations containing *M. oleifera* silage replacing concentrate mixture at different levels in the presence of three levels of *C. vulgaris* in the diets

Replacement^1^	1% *C. vulgaris*							2% *C. vulgaris*							3% *C. vulgaris*						
	DM	OM	CP	EE	NSC	NDF	ADF	DM	OM	CP	EE	NSC	NDF	ADF	DM	OM	CP	EE	NSC	NDF	ADF
0% (control)^2^	78.6	88.3	16.4	4.0	34.5	33.3	20.0	78.6	88.3	16.4	4.0	34.5	33.3	20.0	78.6	88.3	16.4	4.0	34.5	33.3	20.0
0%	78.7	88.3	16.8	4.1	34.2	33.1	19.9	78.8	88.4	17.3	4.2	33.9	33.0	19.8	78.9	88.4	17.7	4.3	33.6	32.8	19.8
10%	76.5	88.2	17.4	4.1	33.1	33.5	20.9	76.6	88.2	17.8	4.2	32.8	33.4	20.8	76.8	88.3	18.2	4.3	32.5	33.2	20.7
20%	74.3	88.0	18.0	4.2	31.9	33.9	21.9	74.5	88.1	18.4	4.2	31.7	33.8	21.8	74.7	88.1	18.8	4.3	31.4	33.6	21.7
30%	72.1	87.9	18.6	4.2	30.8	34.3	22.8	72.3	88.0	19.0	4.3	30.6	34.1	22.7	72.6	88.0	19.4	4.4	30.3	34.0	22.6
40%	69.9	87.8	19.2	4.2	29.7	34.7	23.8	70.2	87.8	19.6	4.3	29.5	34.5	23.7	70.5	87.9	19.9	4.4	29.2	34.3	23.5
50%	67.7	87.6	19.8	4.2	28.5	35.1	24.8	68.0	87.7	20.1	4.3	28.3	34.9	24.6	68.4	87.7	20.5	4.4	28.1	34.7	24.5
60%	65.5	87.5	20.4	4.2	27.4	35.5	25.8	65.9	87.5	20.7	4.3	27.2	35.3	25.6	66.3	87.6	21.1	4.4	27.1	35.1	25.4
70%	63.3	87.3	21.0	4.2	26.3	35.9	26.7	63.8	87.4	21.3	4.3	26.1	35.7	26.6	64.2	87.5	21.6	4.4	26.0	35.5	26.4
80%	61.2	87.2	21.5	4.2	25.2	36.3	27.7	61.6	87.3	21.9	4.3	25.0	36.0	27.5	62.1	87.3	22.2	4.4	24.9	35.8	27.3
90%	59.0	87.0	22.1	4.3	24.0	36.6	28.7	59.5	87.1	22.4	4.4	23.9	36.4	28.5	60.0	87.2	22.7	4.4	23.8	36.2	28.2
100%	56.8	86.9	22.7	4.3	22.9	37.0	29.7	57.3	87.0	23.0	4.4	22.8	36.8	29.4	57.9	87.1	23.3	4.5	22.7	36.6	29.2

*DM*, dry matter; *OM*, organic matter; *CP*, crude protein; *EE*, ether extract; *NDF*, neutral detergent fiber; *ADF*, acid detergent fiber; *NSC*, non-structural carbohydrates

^1^Concentrate feed mixture was replaced by *M. oleifera* silage at different levels (0 to 100%, DM basis) in the presence of three levels of *C. vulgaris* (1, 2, and 3%, DM basis) in the diets

^2^No *C. vulgaris* or *M. oleifera* silage included in the ration

### Feed analysis

Samples of *M. oleifera* silage, *C. vulgaris* microalgae, ensiled vegetable and fruit byproducts, and formulated rations were analyzed for DM, ash content after burning the samples in a muffle furnace at 550°C (method ID 942.05), ether extract (EE) content using diethyl ether in a Soxhlet extractor (method ID 920.39), and N content using Kjeldahl method (method ID 954.01) according to AOAC (2005) methods. The concentration of CP in feed ingredients was calculated as *N* × 6.25. Neutral detergent fiber (NDF) content was determined following the procedure of Van Soest et al. (1991) using sodium sulfite without alpha amylase. Acid detergent fiber (ADF; method ID 973.18) concentration was analyzed and expressed exclusive of residual ash according to AOAC (2005) (method ID 973.18). Non-structural carbohydrate (NSC) [100 – NDF – CP – EE – ash] and OM [100 – ash] contents were calculated.

Tannin contents in *M. oleifera* silage and fresh leaves with smaller twigs were determined according to Makkar (2003) and total phenolic concentration according to Meier et al. (1988). Before the evaluation, the quality of silages was assessed for pH, NH_3_-N, and volatile fatty acids (VFA). A homogenized sample of silage (200 g fresh weight) is mixed with 800 mL of distilled water and homogenized for 3 min with a laboratory blender and then filtrated through 4 layers of cheesecloth. The pH value was measured by using an HI 9321 microprocessor pH/mV/°C bench meter (Hanna® Instrument, Singapore). Ammonia-N concentration was determined by Kjeldahl distillation procedure according to AOAC (2005) (method 941.04). For determination of VFA concentration, a sample (40 mL silage fluid) was centrifuged for 15 min at 6000 ×g at 4°C after the addition of 1 mL metaphosphoric acid solution (25%) to prevent loss of volatiles before total VFA analysis by steam distillation and titration method (2005).

Aflatoxin (B_1_) concentration was measured in *M. oleifera* silage and ensiled vegetable and fruit byproducts using a fluorometer (Series-4, VICAM, Milford, MA, USA) based on the methods described by AOAC (2005).

### In vitro fermentation and biodegradation

In vitro ruminal fermentation was performed using 250-mL bottles (ANKOM^RF^ Gas Production System) fitted with an automatic wireless gas production module (Ankom Technology, Macedon, NY, USA) and pressure sensors. Each gas production module sends measurements via a receiver to an attached computer. The incubation medium containing buffer, macromineral, micromineral, and resazurin solutions and distilled water was prepared according to Goering and Van Soest (1970) in a volumetric flask and flushed continuously with CO_2_ for 2 h at 39°C. A reduction agent (sodium sulfide solution) was added (2 mL) to the buffer shortly before ruminal fluid addition. The ruminal inoculum (20 mL) and the buffer (80 mL) were mixed in each 250-mL bottle and flushed with CO_2_, closed with the module head, and incubated in a thermoshaker with 40 rotations per minute at 39 °C for 48 h. The initial pH of the inoculum was 6.8–6.9.

Rumen inoculum was collected from the rumen of three sheep from a local slaughterhouse at Cairo (Egypt). Before slaughtering, sheep were ad libitum fed a diet containing concentrates, berseem hay, and rice straw at 500:400:100 (DM basis), with free access to water. Rumen contents were collected in a thermos preheated at 39°C and transport to the laboratory where it was flushed with CO_2_. The ruminal fluid was filtered through two-layered cheesecloth and then the particulate materials were squeezed to obtain microbes loosely attached to feed particles.

Individual ingredients were dried, milled (1-mm screen), and mixed before ration formulation. Rations were tested in two 48-h incubation runs with three replicates in each run with 2 bottles containing inoculum but no feed (blanks). A 1 g ±10 mg sample for each diet was weighed into filter bags (ANKOM F57; Ankom Technology, Macedon, NY, USA) and the bags were put into 250-mL bottles. The accumulated gas was released automatically when the pressure inside the bottles exceeded 34.47 kPa above the atmospheric pressure. The absolute pressure was recorded every 10 min and cumulative pressure was calculated from the recorded values.

The pressure of the accumulated gas was converted into volume (mL) at standard pressure and temperature (Ebeid et al. 2022). The average gas produced in the blank bottles was subtracted (blank corrected gas production) to get net gas production at 0, 2, 4, 6, 8, 10, 12, 16, 20, 24, 36, and 48 h. At each incubation time, 5 mL of gases was taken from the sampling vent and injected into a Gas-Pro detector (Gas Analyzer CROWCON Model Tetra3, Abingdon, UK) to measure the concentrations of CH_4_ and CO_2_ in the total gas.

The incubation was terminated after 48 h, by swirling the bottles in ice for 5 min. The pH was measured immediately using a pH meter. The filter bags were removed from the bottles and dried in a forced air oven set at 55° C for 48 h. Dry matter, NDF, and ADF degradation were calculated by difference between the initial weight of the dried substrate DM or NDF or ADF and the weight of DM, NDF, or ADF in the dried residue, respectively.

At 48 h, the fluid samples (5 mL) were collected from each bottle in glass tubes. Subsequently, a 3-mL subsample was preserved with 3 mL of 0.2 *M* hydrochloric acid solution for NH_3_-N analysis (method 954.01) according to AOAC (2005) by steam distillation. Another subsample (0.8 mL) was mixed with 0.2 mL of metaphosphoric acid solution (250 g/L) for total VFA analysis. Individual VFA were measured using a chromatography after processing 1.6 mL of strained in vitro fermented ruminal fluid with 0.4 mL of a solution containing 250 g of metaphosphoric acid as described previously.

Another 4 mL of the fermented fluid was mixed with 4 mL of methyl green-formalin-saline solution and stored in a refrigerator at 4 °C until analysis of bacterial and protozoal count following the procedure described by Dehority (1993). The concentration of total bacteria was determined using a Petroff-Hausser counting chamber (Hausser Scientific®, 3900, Horsham, PA) and a phase contrast microscope at a magnification of 100×. Exactly 0.5 mL of formaldehyde fixed sample was diluted with 4.5 mL of distilled water. The mean concentration of bacteria in fermentation fluid was determined as the average bacterial count in each grid, multiplied by the dilution factors and the chamber factor (2×10^7^).

For the protozoal enumeration, 4 mL of methyl green-formalin-saline solution fixed sample was diluted with 1 mL of distilled water, and then 0.5-mL sample was taken with a Pasteur pipette (BRAND, 7712, Wertheim, Germany) and put into a Neubauer chamber (BRAND, 7178-10, Wertheim, Germany), and observed on a contrast microscope at a 400× magnification. The protozoa were counted in eight quadrants (4 in each grid). The concentration of protozoa of culture medium was calculated as the average protozoal number in each grid, multiplied by the dilution factors and the chamber factor (1×10^4^).

### Gas production kinetics and statistical analyses

Total gas, CO_2_, and CH_4_ production (mL/g DM) kinetic were estimated using the NLIN procedure of SAS (Version 9.4, SAS Inst., Inc., Cary, NC) according to France et al. (2000) model as follows: *y* = *b* × [1 − *e*^−c (t−Lag)^] where *y* is the volume of total gas or CO_2_ or CH_4_ production (mL/g DM) at time *t* (h); *b* is the asymptotic total gas or CO_2_ or CH_4_ production (mL/g DM); *c* is the fractional rate of gas production (/h); and *Lag* (h) is the discrete lag time prior to any gas production.

Data were analyzed using the GLM procedure of SAS (SAS Inst. Inc. Cary, NC, USA) in a complete randomized design using the model: *Y*_ijk_ = *μ* + *R*_i_ + *D*_j_ + (*R* × *D*)_ij_ + *ε*_ijk_ where *Y*_ijk_ is the observation, *μ* is the population mean, *R*_i_ is the ration type effect, *D*_j_ is the replacement level effect, (*R* × *D*)_ij_ is the interaction between ration type and replacement level, and *ε*_ijk_ is the residual error. One-way ANOVA was also performed within each level of *C. vulgaris* including the control diet. When ANOVA was significant, Dunnett test was performed to find out the significant effect compared with the control. Linear and quadratic contrasts were used to examine dose responses to increasing replacement levels.

## Results

### Chemical composition

The fresh *M. oleifera* leaves contained about 29% CP, 21% NSC, and 35% NDF, while the ensiled *M. oleifera* leaves contained about 28% CP, 19% NSC, and 35% NDF (Table 1). The basal concentrate mixture (without *M. oleifera* silage or *C. vulgaris*) contained 16% CP and 27% NDF. The *C. vulgaris* microalgae contained high CP (58%) and low (12%) NDF.

Increasing the replacement level of concentrate mixture by *M. oleifera* silage gradually decreased OM and NSC and gradually increased CP, NDF, and ADF concentrations (Table 3). Increasing the level of *C. vulgaris* microalgae in rations gradually increased the concentration of CP with slight effects on other nutrients.

### Biogas production

**Fig. 1.**
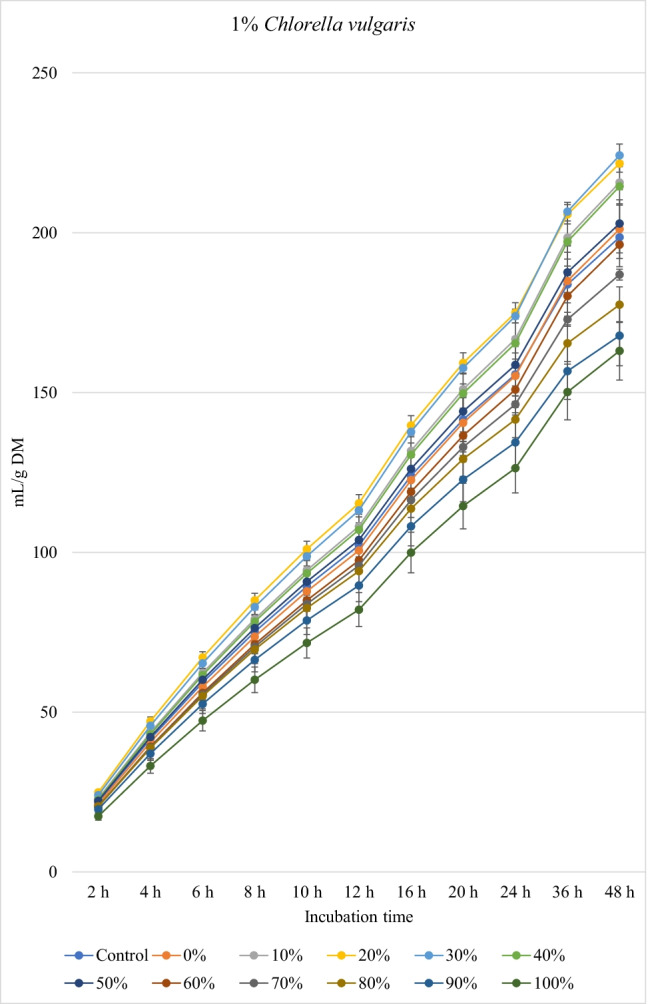

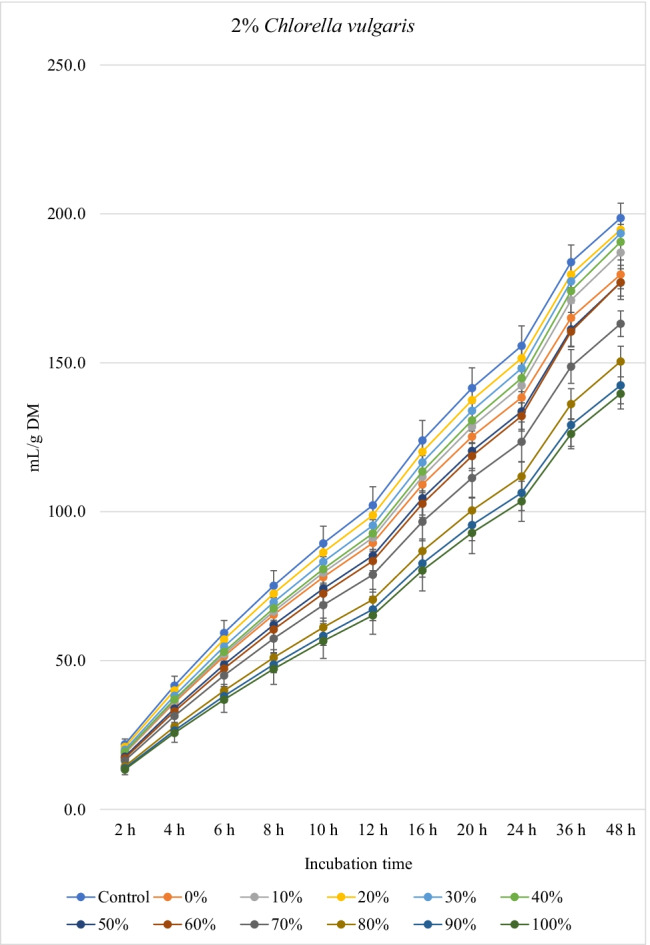

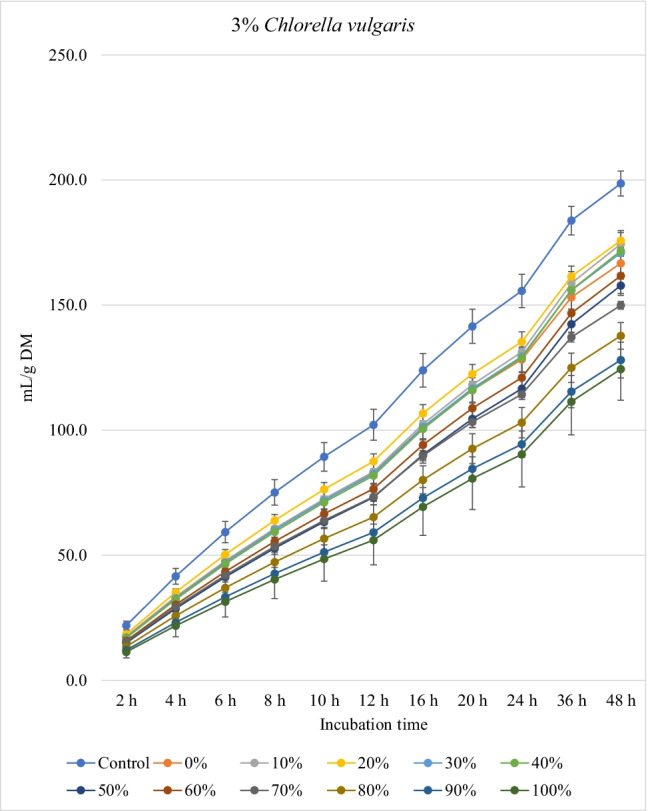
In vitro ruminal total gas production from rations containing *M. oleifera* silage replacing concentrate feed mixture at 10 different levels (0 to 100%, basis DM) in the presence of three levels *C. vulgaris* (1, 2, and 3% DM basis) in the diets (*P* values: *M. oleifera* <0.001, *C. vulgaris* <0.001, *M. oleifera* × *C. vulgaris* = 0.710). Control = No *C. vulgaris* or *M. oleifera* included in the rations

**Fig. 2.**
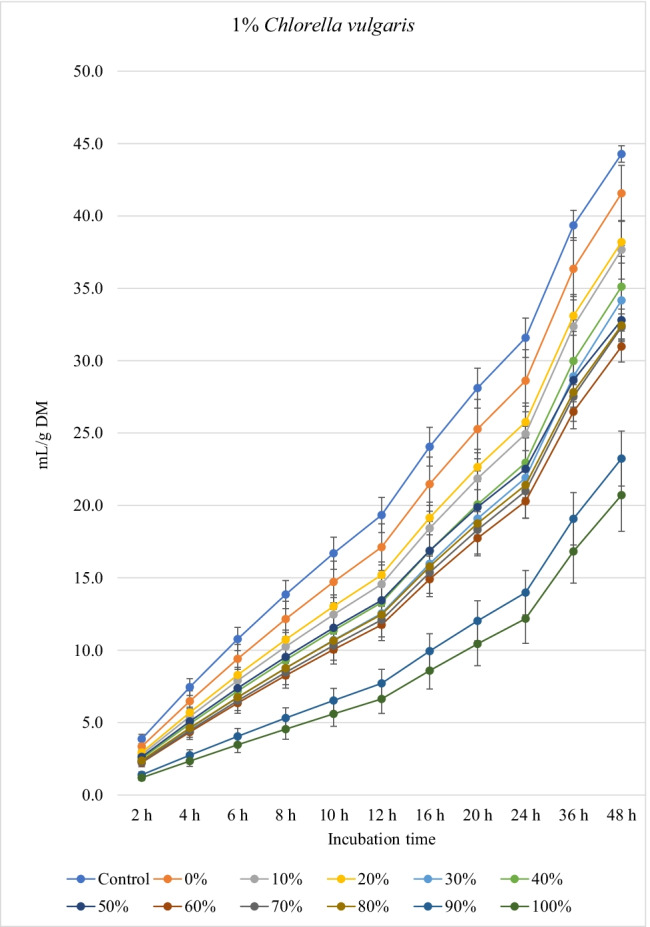

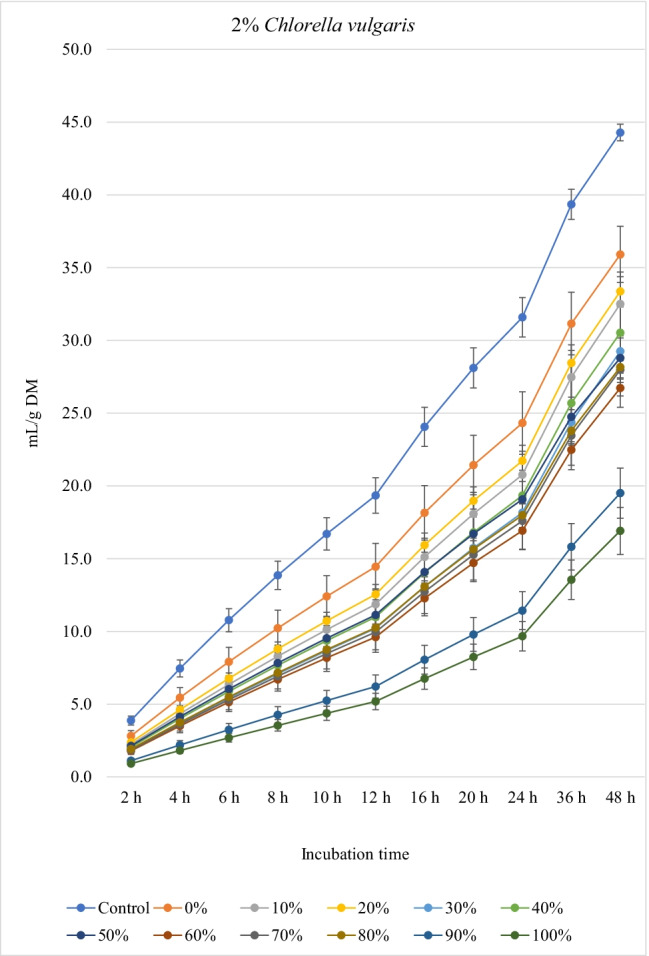

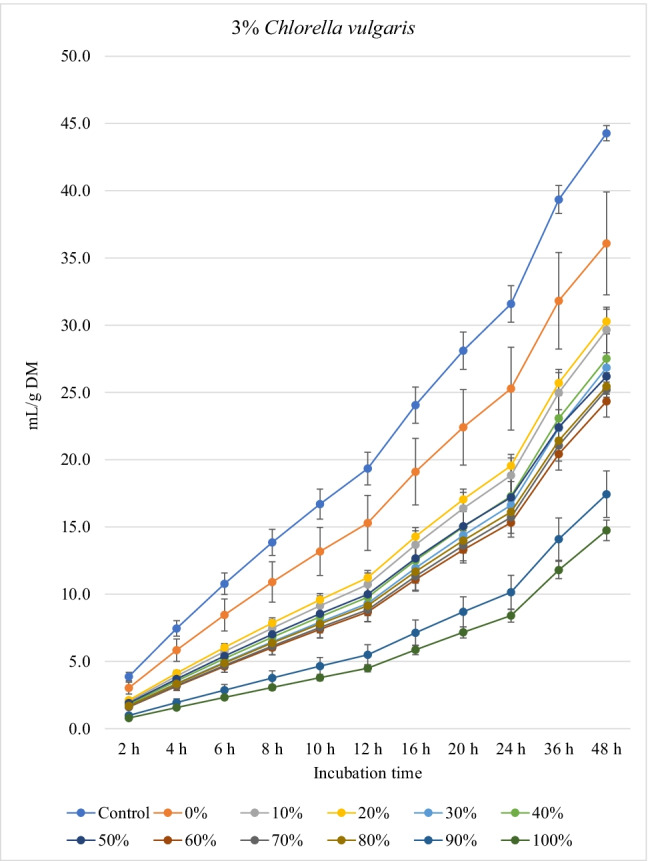
In vitro ruminal methane production from rations containing *M. oleifera* silage replacing concentrate feed mixture at 10 different levels (0 to 100%, basis DM) in the presence of three levels *C. vulgaris* (1, 2, and 3% DM basis) in the diets (*P* values: *M. oleifera* silage <0.001, *C. vulgaris* <0.001, *M. oleifera* × *C. vulgaris* = 0.998). Control = No *C. vulgaris* or *M. oleifera* included in the rations

**Fig. 3.**
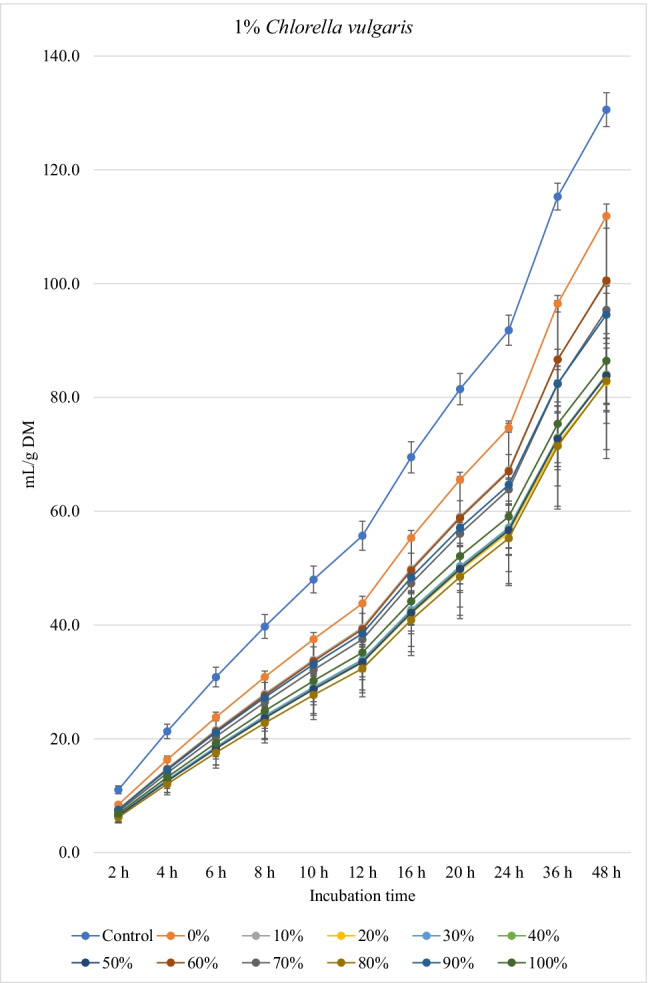

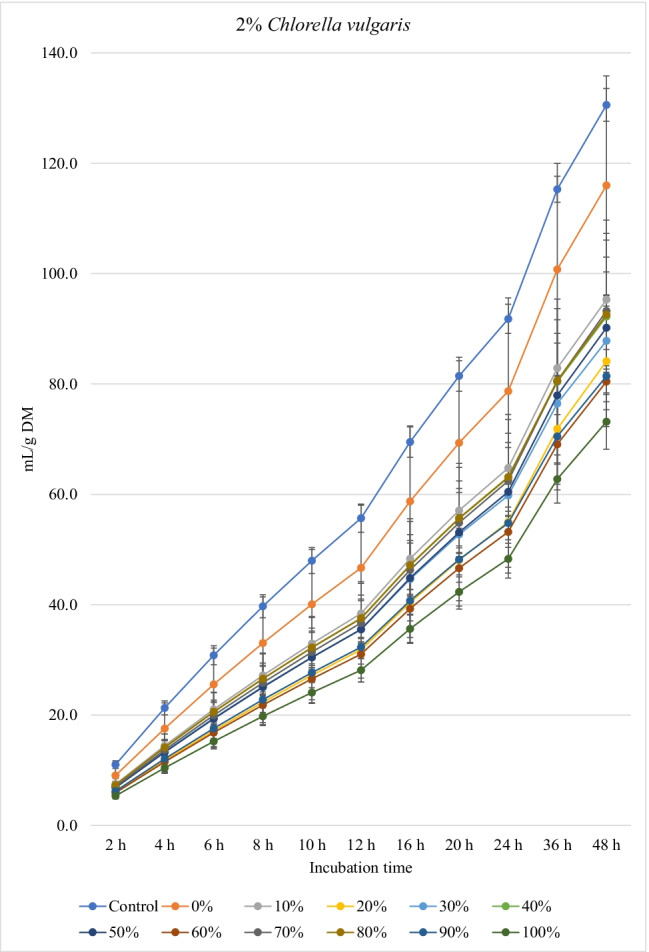

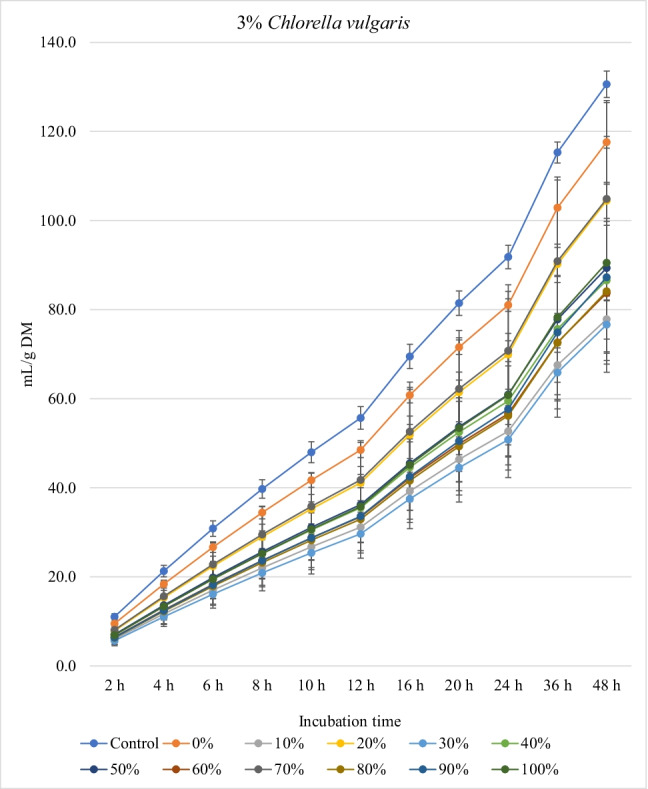
In vitro ruminal carbon dioxide production from rations containing *M. oleifera* silage replacing concentrate feed mixture at 10 different levels (0 to 100%, basis DM) in the presence of three levels of *C. vulgaris* (1, 2, and 3% DM basis) in the diet (*P* values: *M. oleifera* silage <0.001, *C. vulgaris* = 0.118, *M. oleifera* × *C. vulgaris* = 0.191). Control = No *C. vulgaris* or *M. oleifera* included in the rations

Figures 1, 2, and 3 show the in vitro ruminal total gas, CH_4_, and CO_2_ production (mL/g incubated DM), respectively, from ration containing different levels of *M. oleifera* silage replacing concentrate mixture in the presence of *C. vulgaris* microalgae at different incubation times. For the kinetics of total gas, CH_4_, and CO_2_ production, no *M. oleifera* × *C. vulgaris* microalgae interactions were observed; however, significant interactions were observed for rate of total gas production and lag time for CH_4_. Replacement of concentrates with *M. oleifera* silage affected the asymptotic total gas, CH_4_, and CO_2_ production; the rate of total gas and CH_4_ production; total gas, CH_4_, and CO_2_ production at 48 h; and the lag time of CH_4_ production, while *C. vulgaris* microalgae levels affected the asymptotic total gas and CH_4_ productions; the rate of total gas and CH_4_ production; the lag time of total gas and CH_4_ production; and total gas and CH_4_ and CO_2_ volume at 48 h (Table 4).

**Table 4:** In vitro ruminal total gas, methane (CH_4_), and carbon dioxide (CO_2_) production (mL/g DM) and kinetics of rations containing *M. oleifera* silage replacing concentrate feed mixture at different levels (0 to 100%, DM basis) in the presence of three levels of *C. vulgaris* microalgae (1, 2, and 3%, DM basis) in the diets

Ration^1^	Replacement level	Gas production^3^				CH_4_ production^3^				CO_2_ production^3^			
		*b*	*c*	*Lag*	Total gas	*b*	*c*	*Lag*	Total CH_4_	*b*	*c*	*Lag*	Total CO_2_
Control^2^	0%	215	0.054	1.61	199	53.0	0.038	1.60	44.3	160	0.036	1.63	131
1% *C. vulgaris* microalgae	0%	221	0.051	1.88*	201	52.5	0.033	1.57	41.6	149	0.030	1.82	112*
	10%	236*	0.051	1.86*	216*	51.2	0.028*	1.67	37.7*	134*	0.027*	1.81	100*
	20%	239*	0.055	1.86*	222*	49.8	0.030*	1.71	38.2*	107*	0.024*	1.71	82.7*
	30%	245*	0.052	1.79*	224*	50.4	0.024*	1.69	34.2*	108*	0.024*	1.74	83.8*
	40%	235*	0.051	1.87*	215*	49.1	0.026*	1.62	35.1*	109*	0.024*	1.82	84.0*
	50%	220	0.053	1.80*	203	41.5*	0.033	1.74	32.8*	109*	0.024*	1.87	83.8*
	60%	216	0.053	1.84*	196	43.1*	0.027*	1.87	31.0*	134*	0.026*	1.84	101*
	70%	203	0.051	1.78*	187*	45.8*	0.026*	1.74	32.3*	126*	0.025*	1.84	95.4*
	80%	190*	0.050*	1.85*	178*	44.2*	0.028*	1.65	32.4*	111*	0.024*	1.82	82.9*
	90%	179*	0.050*	1.87*	168*	42.0*	0.017*	1.85	23.2*	121*	0.025*	1.83	94.6*
	100%	178*	0.051	1.81*	163*	41.1*	0.015*	1.59	20.7*	110*	0.024*	1.77	86.5*
	SEM	3.35	0.0013	0.062	3.64	0.92	0.0018	0.068	1.13	7.27	0.0014	0.098	5.42
	Linear	<0.001	0.008	0.044	<0.001	<0.001	<0.001	0.412	<0.001	0.037	0.037	0.608	0.066
	Quadratic	<0.001	0.893	0.514	<0.001	0.102	0.003	0.303	0.010	0.026	1.000	0.815	0.018
2% *C. vulgaris* microalgae	0%	197	0.050	1.73	180*	47.7	0.030	1.59	35.9*	152	0.035	1.69	116*
	10%	207	0.048	1.70	187*	48.1	0.024*	1.51	32.5*	123*	0.025	1.76	95.3*
	20%	212	0.052	1.68	195	46.8	0.026*	1.73	33.4*	118*	0.025	1.71	84.1*
	30%	214	0.049	1.69	194	47.4	0.020*	1.59	29.2*	113*	0.024	1.54	87.8*
	40%	212	0.048	1.63	191	46.2	0.023*	1.69	30.5*	118*	0.024	1.87	92.2*
	50%	198*	0.047*	1.80*	177*	39.0*	0.028*	1.69	28.8*	119*	0.024	1.71	90.2*
	60%	200*	0.045*	1.64	177*	40.5*	0.023*	1.68	26.7*	110*	0.023	1.63	80.5*
	70%	182*	0.047*	1.71	163*	43.0*	0.022*	1.69	28.0*	123*	0.025	1.81	93.3*
	80%	171*	0.044*	1.76	150*	41.5*	0.024*	1.63	28.2*	119*	0.024	1.53	92.6*
	90%	161*	0.045*	1.74	142*	39.5*	0.014*	1.65	19.5*	107*	0.023	1.87	81.4*
	100%	160*	0.044*	1.79*	140*	38.6*	0.012*	1.64	16.9*	99.6*	0.023	1.79	73.2*
	SEM	3.25	0.0019	0.092	2.89	0.89	0.0022	0.099	1.35	7.73	0.0022	0.078	6.75
	Linear	<0.001	0.001	0.045	<0.001	<0.001	<0.001	0.064	<0.001	0.001	0.780	0.415	0.002
	Quadratic	<0.001	0.878	0.456	<0.001	0.481	0.055	0.111	0.015	0.279	0.689	0.348	0.377
3% *C. vulgaris *microalgae	0%	184*	0.050	1.88*	167*	44.2*	0.035	1.65	36.1	148	0.033	1.73	117.6
	10%	196*	0.046*	1.90*	174*	44.5*	0.023*	1.70*	29.7*	101*	0.030	1.78	77.8
	20%	193*	0.050	1.80*	176*	43.3*	0.025*	1.69*	30.3*	138	0.030	1.82	105
	30%	191*	0.047*	1.58	171*	43.8*	0.020*	1.65	26.8*	104*	0.027	1.74	76.6
	40%	193*	0.046*	1.71*	172*	42.7*	0.022*	1.63	27.5*	109*	0.030	1.66	86.6
	50%	181*	0.043*	1.75*	158*	36.1*	0.027*	1.68*	26.2*	115*	0.030	1.69	89.4
	60%	183*	0.045*	1.91*	162*	37.5*	0.022*	1.60	24.3*	109*	0.033	1.63	83.7
	70%	166*	0.049	1.94*	150*	39.8*	0.021*	1.61	25.2*	137	0.030	1.68	105
	80%	156*	0.045*	1.92*	138*	38.4*	0.023*	1.65	25.4*	112*	0.030	1.73	84.1
	90%	147*	0.043*	1.91*	128*	36.5*	0.014*	1.78*	17.4*	119*	0.027	1.60	87.3
	100%	146*	0.040*	1.93*	124*	34.4*	0.012*	1.66	14.8*	119*	0.030	1.71	90.5
	SEM	3.42	0.0018	0.077	3.62	1.00	0.0014	0.077	1.10	10.8*	0.0020	0.109	8.66
	Linear	<0.001	0.004	0.068	<0.001	<0.001	<0.001	0.015	<0.001	0.455	0.353	0.276	0.260
	Quadratic	<0.001	0.553	0.037	<0.001	0.973	0.458	0.176	0.122	0.009	0.737	0.537	0.122
SEM		3.34	0.0017	0.078	3.40	0.94	0.0018	0.082	0.80	8.67	0.0020	0.096	6.97
*P* value													
*M. oleifera* level		<0.001	0.004	0.252	<0.001	<0.001	<0.001	0.002	<0.001	<0.001	0.835	0.457	<0.001
Microalgae level		<0.001	<0.001	0.002	<0.001	<0.001	<0.001	<0.001	<0.001	0.747	0.127	0.075	0.260
*M. oleifera* × microalgae		0.310	0.006	0.077	0.755	1.000	0.981	0.012	1.000	0.181	0.647	0.288	0.180

*Mean data is significantly different from control within each *C. vulgaris* microalgae level

^1^Concentrate feed mixture was replaced by *M. oleifera* silage at different levels (0 to 100%, DM basis) in the presence of three levels of *C. vulgaris* (1, 2, and 3%, DM basis) in the diets

^2^No *C. vulgaris* or *M. oleifera* silage included in the ration

^3^*b* is the asymptotic total gas or methane or carbon dioxide production (mL/g DM); *c* is the rate of total gas or methane or carbon dioxide production (/h); *Lag* time is the initial delay before total gas or methane or carbon dioxide production starts (h)

For the rations containing 1% *C. vulgaris* microalgae, the asymptotic total gas production and total amount of produced gases showed linear (*P*<0.01) and quadratic (*P*<0.01) responses with increasing replacements of concentrate with *M. oleifera* with gradual increases from 10 to 40% level and thereafter gradually decreases up to 100% levels. All rations containing *M. oleifera* linearly increased the lag time of total gas production (*P*<0.05) compared to the control ration. Rations containing *M. oleifera* linearly decreased the asymptotic CH_4_ and CO_2_ production, total gas, and the rate of CH_4_ and CO_2_ production (*P*<0.05) without affecting their lag time.

For the ration containing 2% *C. vulgaris* microalgae, replacing concentrate with *M. oleifera* linearly and quadratically (*P*<0.05) decreased the asymptotic total gas, total produced amounts of gas, and CH_4_, and linearly (*P*<0.01) decreased CO_2_ production, rate of total gas and CH_4_ production, and total produced amounts of CO_2_ at 48 h, while linearly increased (*P*<0.05) the lag time of total gas production without affecting the lag time of CH_4_ and CO_2_ or the rate of CO_2_ production.

For the ration containing 3% *C. vulgaris* microalgae, increasing levels of *M. oleifera* in diets linearly (*P*<0.01) decreased the asymptotic total gas, CH_4_, and CO_2_ productions; rate of total gas, CH_4_, and CO_2_ production; total gas, CH_4_, and CO_2_ production at 48 h; and the lag time of CH_4_ production (with the replacement levels from 30 to 50%) without affecting the lag time and rate of CO_2_ production.

### Degradability and fermentation

*M. oleifera* × *C. vulgaris* microalgae interactions were observed (*P*<0.05) for ruminal pH and the concentrations of NH_3_-N, total VFA, and propionate. Degradabilities of DM, NDF, and ADF and the concentrations of ruminal NH_3_-N, total VFA, acetate, and propionate differed (*P*<0.05) among rations with different levels of *M. oleifera* and *C. vulgaris* microalgae (Table 5).

**Table 5:** In vitro degradability, ruminal fermentation profile, and bacterial and protozoa counts in rations containing *M. oleifera* silage replacing concentrate feed mixture at different levels (0 to 100% of DM) in the presence of three levels of *C. vulgaris* microalgae (1, 2, and 3% of mixture, DM) in the diets

Ration^1^	Replacement level	Degradability^3^			Ruminal microorganisms^4^		Fermentation^5^		Volatile fatty acids^6^			
		DM	NDF	ADF	Bacteria	Protozoa	Ammonia-N	pH	Total	Acetate	Propionate	Butyrate
Control^2^	0%	59.4	55.6	49.3	12.6	6.14	11.0	6.00	56.9	34.0	13.1	9.79
1% *C. vulgaris *microalgae	0%	62.3	59.5	54.2*	13.8	4.13	13.5*	5.94	59.9	35.2	15.4	9.35
	10%	63.1	61.8	51.7	13.9	4.47	13.2*	6.17	62.5	37.3	16.2*	8.98
	20%	63.0	62.9	52.8	13.7	4.49	14.3*	6.23	61.8	36.7	14.8	10.38
	30%	66.1*	63.6*	54.0*	15.6*	4.07	14.4*	6.00	69.5*	42.1*	18.2*	9.13
	40%	61.5	58.8	51.7	14.4	4.09	14.3*	5.79	64.4*	39.4*	15.7	9.28
	50%	59.8	58.3	51.8	14.3	4.24	14.0*	5.88	59.5	34.6	15.2	9.72
	60%	59.7	59.0	52.2	13.5	4.50	14.7*	6.07	64.3*	38.8	16.0*	9.57
	70%	55.9	60.1	51.3	13.9	4.73	15.2*	6.03	61.0	36.3	15.2	9.58
	80%	55.5	60.7	50.5	13.7	4.12	14.4*	6.32	59.5	36.5	14.7	8.40
	90%	53.8*	61.1	50.1	13.3	4.64	14.4*	5.95	60.6	35.9	15.0	9.68
	100%	55.6*	58.2	49.9	13.9	5.01	15.0*	6.04	67.0*	38.8	18.9*	9.35
	SEM	1.43	2.56	1.78	0.54	0.654	0.29	0.153	1.69	1.50	0.72	0.440
	Linear	<0.001	0.040	0.042	0.028	0.415	<0.001	0.866	0.033	0.963	0.001	0.674
	Quadratic	0.112	0.932	0.771	0.041	0.583	0.116	0.663	0.046	0.028	0.124	0.684
2% *C. vulgaris* microalgae	0%	58.4	52.2	50.4	12.4	5.12	11.8	6.11	59.8	35.8	14.0	10.01
	10%	61.3	62.7	52.3	12.5	4.83	12.9	6.22	61.3	37.5	14.3	9.60
	20%	62.2	60.7	48.7	11.9	4.56	13.4	6.46	64.8	38.4	17.4	9.00
	30%	62.7	63.4	50.5	13.9*	4.19*	13.2	6.55	68.8*	43.0*	16.2*	9.50
	40%	59.8	57.1	48.5	12.3	4.85	13.9	6.00	69.4*	41.9*	17.7*	9.74
	50%	56.7	54.9	47.4	13.4*	4.94	14.8*	6.23	62.2	38.2	15.0	8.97
	60%	55.2	54.7	47.4	13.4*	5.05	14.8*	6.14	66.1*	40.0*	16.2*	9.94
	70%	56.5	58.0	47.7	12.7	4.82	14.6*	6.11	61.9	37.9	14.3	9.68
	80%	54.6*	55.6	44.3*	13.3	4.62	14.6*	6.28	63.9	39.2*	15.8*	8.98
	90%	54.0*	58.4	45.9	13.1	4.30*	14.6*	6.04	61.4	36.0	15.7*	9.64
	100%	52.5*	55.6	42.6*	13.2	4.78	15.2*	5.98	59.2	35.2	14.9	9.09
	SEM	1.6	1.6	2.1	0.4	0.423	0.3	0.163	1.9	1.9	0.6	0.465
	Linear	<0.001	0.099	0.001	0.027	0.035	<0.001	0.134	0.004	0.004	0.002	0.429
	Quadratic	0.098	0.241	0.587	0.296	0.903	0.006	0.273	0.271	0.352	0.992	0.882
3% *C. vulgaris *microalgae	0%	51.2*	51.1	46.3	11.0*	4.58*	12.9*	5.7	56.6	35.0	12.4	9.17
	10%	50.8*	52.1	46.0	11.4*	5.20*	13.6*	6.13	59.1	36.3	13.9	8.87
	20%	55.5	53.7	46.3	11.8	4.74*	13.8*	6.04	56.8	33.9	13.9	9.02
	30%	56.1*	53.1	47.9	11.9	4.57*	13.6*	6.25	59.2	34.6	15.7	8.95
	40%	55.1*	53.2	46.3	11.4*	4.71*	14.1*	5.81	56.7	34.1	13.6	9.02
	50%	53.9	50.7*	44.8*	11.5*	5.18*	14.2*	6.37	57.7	34.7	13.7	9.35
	60%	52.9*	52.3	47.1	11.5*	3.16*	14.8*	6.74	58.4	35.8	13.6	8.95
	70%	51.2*	53.6	44.1*	11.2*	4.12*	14.7*	6.60	56.7	35.0	12.7	8.98
	80%	51.8*	50.1*	45.1	11.2*	4.37*	14.4*	6.76	56.2	34.8	12.6	8.76
	90%	50.0*	52.1	44.7*	11.1*	4.12*	14.9*	6.74	56.9	35.9	12.2	8.80
	100%	47.7*	49.5*	41.5*	12.1	3.77*	14.8*	6.44	56.8	34.4	12.6	9.79
	SEM	2.04	1.5	1.7	0.4	0.440	0.2	0.222	0.8	0.7	0.3	0.334
	Linear	0.008	0.025	0.042	0.009	0.017	<0.001	0.090	0.140	0.935	0.301	0.564
	Quadratic	0.054	0.151	0.180	0.006	0.835	0.065	0.218	0.326	0.517	0.111	0.326
SEM		1.70	1.95	1.90	0.44	0.517	0.27	0.160	1.54	1.44	0.56	0.417
*P* value												
*M. oleifera* level		<0.001	0.002	0.007	0.010	0.931	<0.001	0.486	<0.001	0.007	<0.001	0.548
Microalgae level		<0.001	<0.001	<0.001	<0.001	0.247	0.033	<0.001	<0.001	<0.001	<0.001	0.055
*M. oleifera* × microalgae		0.504	0.786	0.987	0.143	0.701	0.009	0.015	0.010	0.163	<0.001	0.527

*Mean data is significantly different from control within each *C. vulgaris* microalgae level

^1^Concentrate feed mixture was replaced by *M. oleifera* silage at different levels (0 to 100%, DM basis) in the presence of three levels of *C. vulgaris* (1, 2, and 3%, DM basis) in the diets

^2^No *C. vulgaris* or *M. oleifera* silage included in the rations

^3^Degraded substrate (%), *DM* is dry matter, *NDF* is neutral detergent fiber, and *ADF* is acid detergent fiber

^4^Ruminal microorganisms (number per mL incubation medium): bacteria (total count × 10^8^) and protozoa (total count × 10^5^)

^5^Ammonia-N (mg/dL)

^6^Volatile fatty acids concentration (mmol/L)

For the rations containing 1% *C. vulgaris* microalgae, the replacement level of 30% showed the highest DM and ADF degradabilities (*P*<0.05), while the levels from 70 to 100% decreased DM degradability (*P*<0.01) compared to the control ration. All rations containing *M. oleifera* linearly increased (*P*<0.05) NDF degradability, ruminal bacteria count, and the concentrations of ruminal NH_3_-N and propionate, while decreasing ruminal protozoal count (*P*<0.05).

For the rations containing 2% *C. vulgaris* microalgae, the replacement levels of 20 and 30% increased DM degradability, while the replacement levels from 50 to 100% decreased it (*P*<0.05). Moreover, the replacement levels from 80 to 100% decreased ADF degradability, whereas all replacement levels did not affect NDF degradability (*P*<0.05). Rations containing *M. oleifera* linearly decreased (*P*<0.05) ruminal protozoal count, and linearly increased (*P*<0.05) the concentrations of ruminal NH_3_-N, total VFA, acetate, and propionate.

For the rations containing 3% *C. vulgaris* microalgae, replacing concentrate with *M. oleifera* linearly decreased (*P*<0.05) DM and NDF degradabilities and ruminal bacterial and protozoal counts, but increased ruminal NH_3_-N concentration (*P*<0.05) without affecting total or individual VFA concentrations.

## Discussion

Because of the insignificant interactions between *M. oleifera* × *C. vulgaris* for most measured parameters, their effects will be discussed individually. However, significant interactions for the variables will be briefly discussed.

### Biogas production

The significant *M. oleifera* × *C. vulgaris* interaction for rate of gas production revealed the rate of gas production at 2% and 3% levels of *C. vulgaris* to be reduced with increasing doses of *M. oleifera* silage, but not at 1% level of *C. vulgaris*, which might be attributed to the presence of inhibitory principles present in microalgae (e.g., higher content of unsaturated fatty acids and rigid cell wall) and moringa (e.g., phenolics), both of which at greater levels caused lower fermentation rate*.* Thus, it indicates that the rate of gas production is a matrix of *M. oleifera* and *C. vulgaris* levels, and the level of *C. vulgaris* should be considered for each *M. oleifera* level (replacement level). Levels of *M. oleifera* affected total gas, CH_4_, and CO_2_ production kinetics of many variables, probably due to the differences of their chemical composition especially fiber (NDF, ADF, and lignin) and NSC contents and plant bioactive compounds. *C. vulgaris* at 1% showed better results (e.g., higher gas production and nutrient degradability, lower CH_4_ and CO_2_ production) compared to the other levels of *C. vulgaris*. Inclusion of *C. vulgaris* at 2% to the diet (25% concentrate and 75% corn silage) increased gas production, which indicates enhanced microbial activity in the rumen (Dubois et al. 2013). *C. vulgaris* is reported to contain a unique phytonutrient known as *C. vulgaris* CGF, which comprises of nucleic acid associated with amino acids, peptides, proteins, vitamins, and sugars, and it improves growth of bacteria (Kotrbáček et al. 2015; Kholif and Olafadehan 2021b). Additionally, β-glucan is present in *C. vulgaris*, which can scavenge free radicals (Iwamoto 2004), thus improving ruminal fermentation (Kholif and Olafadehan 2021b). These positive effects on ruminal fermentation were observed with the low level of *C. vulgaris* compared to the other levels (i.e., 2 and 3% *C. vulgaris*). These results confirm the results observed by Kholif et al. (2017) who stated negative effects on in vitro ruminal fermentation due to increasing inclusion levels of *C. vulgaris*. For the rations containing 1% *C. vulgaris*, *M. oleifera* replacing concentrate at 10 to 40% increased the asymptotic gas production; however, high replacement levels (e.g., 80 to 100%) decreased the asymptotic gas production which may be due to increasing concentrations of antinutritional factors in *M. oleifera*. Astutia et al. (2011) observed that *M. oleifera* leaf supplementation at 30% of diets of sheep optimized rumen fermentation. A vivo meta-analysis study also revealed that supplementation of tree leaves up to 40% of the diets could improve feed digestibility and ruminal fermentation in sheep (Patra 2010). Low levels of secondary metabolites can be used by ruminal microbiota as energy sources (Kholif and Olafadehan 2021a). Additionally, the presence of secondary phenolic metabolites in *M. oleifera* extracts may provide strong free radicals scavenging activity and lipid peroxidation inhibition properties. Higher gas production at low replacement levels may be attributed to greater substrate degradation due to the phytochemicals present in *M. oleifera*. Secondary metabolites present in many plants and herbs have been reported to stimulate fibrolytic microbial activities in the rumen (Morgavi et al. 2000) leading to faster rate of fermentation and degradation of substrates (Kholif and Olafadehan 2021a). Antioxidant properties have been suggested to increase microbial activities in the rumen by ameliorating oxidative insults of the anaerobic microbiota (Singla et al. 2021) and *M. oleifera* leaves have a high antioxidant action (IC50 49.86 μg/mL) (Kashyap et al. 2022) that can enhance substrate degradability.

The negative effects of high replacement levels may be attributed to increasing the concentrations of secondary metabolites (e.g., tannins, saponins, and flavonoids), which can inhibit rumen microbes at high concentrations in rations (Kholif and Olafadehan 2021a). Additionally, *M. oleifera* in the ration containing 1 and 2% *C. vulgaris* increased the lag time of gas production, which may be related with the increased fiber contents when *M. oleifera* replaced the concentrates in diets.

The significant *M. oleifera* × *C. vulgaris* interaction for the lag of CH_4_ production occurred as a result of greater lag time at 3% *C. vulgaris* along with increasing levels of *M. oleifera* level, which might be due to inhibition of methanogenic activity with high level of microalgae along with moringa silage. Thus, this result suggests that the lag of CH_4_ production is ration- and algae-level-dependent, thus underpinning the importance of identifying optimal supplemental levels of *C. vulgaris* for each ration containing different levels of *M. oleifera*. *M. oleifera* decreased the asymptotic and rates of CH_4_ and CO_2_ production. It was expected that increasing nutrient degradability at low replacement levels (i.e., up to 30–40%) would increase CH_4_ production as a result of the higher fermentation activities and digestion process. However, this was not noted in the present experiment, which may be related to the presence of tannins and saponins in *M. oleifera* silage, because plant secondary compounds such as tannins and saponins inhibit activity of methanogens and decrease ruminal CH_4_ production (Ku-Vera et al. 2020). Phenolic compounds in *M. oleifera* leaves have strong antibacterial effects on some microbial species such as *Staphylococcus aureus*, *Escherichia coli*, and *Salmonella typhi* (Peixoto et al. 2011) and also on CH_4_-producing archaea in the rumen due to the antiprotozoal effects of phenolics (Ku-Vera et al. 2020; Kholif and Olafadehan 2021a). Phenolics disrupt the membrane of rumen archaea and bind the proteinaceous adhesin or parts of the cell envelope, impairing the establishment of the methanogen-protozoa complex, decreasing interspecies hydrogen transfer and inhibition of methanogen growth (Ku-Vera et al. 2020). Decreasing DM degradability with the diets containing high levels of *M. oleifera* silage can partially explain the reduction in CH_4_ production; however, reducing CH_4_ production with *M. oleifera* may not primarily due to the reduction in DM digestibility but associated with the inhibitory effects of *M. oleifera* secondary metabolites on methanogenic activity (Akanmu and Hassen 2018; Ku-Vera et al. 2020). Akanmu and Hassen (2018) observed that the secondary metabolites in *M. oleifera* extract decreased in vitro CH_4_ production at 25 and 50 mg/L distilled water.

*C. vulgaris* independently reduced CH_4_ production in the rumen by 18.5% (at 3% level) compared with the control. *C. vulgaris* is rich in *n*-3 long-chain polyunsaturated fatty acids including eicosapentaenoic and docosahexaenoic contents (Kholif et al. 2017; Madeira et al. 2017) that are strong inhibitors of methanogens and CH_4_ production (Patra and Yu 2013). As protozoal number was not affected by *C. vulgaris*, a decrease in methanogenesis was independent of protozoal contribution, perhaps by direct inhibition of methanogens. Anele et al. (2016) reported negative correlations between CH_4_ production and carbohydrate, oleic acid, and α-linolenic acid content in microalgae. Kholif et al. (2017) comparing different levels of *C. vulgaris* (2, 4, and 8% DM) observed that low levels of microalgae showed better effects on ruminal fermentation than the higher levels. High levels of *C. vulgaris* can act as an antimicrobial agent against ruminal bacteria, protozoa, and fungi, thus causing reduced microbial fermentation activity (Kholif et al. 2017). Many microalgae contain toxic metabolites (e.g., phycotoxins, cyclic peptides, alkaloids, lipopolysaccharides, phenolics, and aromatic compounds) with antibacterial and antifungal properties (Camacho et al. 2007; Janczyk et al. 2009). Such results indicate that an optimal level of *C. vulgaris* could improve ruminal fermentation efficiency, while greater levels depress it.

### Degradability and fermentation

The significant *M. oleifera* × *C. vulgaris* microalgae interactions for ruminal pH and the concentrations of NH_3_-N, total VFA, and propionate indicate a synergy between levels of replacement of concentrate with *M. oleifera* and level of *C. vulgaris* on these parameters. Concentrations of total VFA and propionate at 3% level of microalgae were not affected, but total VFA and propionate concentrations at 1% and 2% of microalgae improved in the presence of moringa silage, indicating lower concentrations of microalgal and moringa bioactives promoted carbohydrate fermentation by ruminal microorganisms. Ammonia concentrations increased more at the higher levels of microalgae, which was likely due to greater concentration of protein along with greater degradation of protein in microalgae. The significant interactions suggest that it is important to identify appropriate *C. vulgaris* level and *M. oleifera* inclusion level in the rations (Kholif et al. 2017). As previously noted, the chemical composition differed between the formulated diets with different levels of *M. oleifera* and *C. vulgaris*. In the ration containing 1% *C. vulgaris*, *M. oleifera* replacing concentrate at 30% increased DM degradability, further confirming that 30% replacement level is the best level of replacement when *C. vulgaris* is used at 1% of the diet. Abdel-Raheem and Hassan (2021) observed that replacing soybean with *M. oleifera* leaf meal at 50 and 75% in buffalo calves diets improved DM, OM, and fiber digestibility, while decreased CP and EE digestibility. As previously mentioned, secondary metabolites and antioxidant properties present in *M. oleifera*, at appropriate levels, can stimulate ruminal fibrolytic microbes and microbial growth (Morgavi et al. 2000; Singla et al. 2021) resulting in faster degradation rate and extent of substrates (Kholif and Olafadehan 2021a).

Increasing the replacement level at 70 to 100% (in the rations containing 1% *C. vulgaris*) and at 50 to 100% (in the rations containing 2% *C. vulgaris*) decreased DM degradability. Conversely, *M. oleifera* silage in the ration containing 1% *C. vulgaris* increased NDF degradability while in the ration containing 2% *C. vulgaris* replacing the concentrate with *M. oleifera* at 80 to 100% decreased ADF degradability, indicating that increasing replacement level is not recommended. The observed improvement with the low replacement levels confirms the previous findings by Ebeid et al. (2020) who reported that rumen microbiota can use low levels of secondary metabolites present in *M. oleifera* (e.g., phenolics, essential oils, and saponins) and utilize them as energy sources (Kholif and Olafadehan 2021a). Although the effect of the rations on enzymatic activities was not measured in the present experiment, we can speculate that increasing level of *M. oleifera* leaf silage in diets may reduce the activity of ruminal cellulase, α-amylase, lipase, urease, and protease (Abdel-Raheem and Hassan 2021).

*M. oleifera* in the ration increased ruminal bacteria count revealing that the secondary compounds in *M. oleifera* such as cationic polyelectrolyte proteins were within acceptable range to exhibit beneficial antibacterial responses (Makkar et al. 2007). Rations containing *M. oleifera* and *C. vulgaris* at 1, 2, and 3% linearly decreased ruminal protozoal count, which could be ascribed to the presence of saponins in *M. oleifera*, a well-documented antiprotozoal agent (Patra and Saxena 2009; Ebeid et al. 2020). Additionally, the presence of unsaturated fatty acids in *M. oleifera* can be considered a toxic material to ciliated protozoa (Ebeid et al. 2020).

Overall, *M. oleifera* in the ration containing *C. vulgaris* at all levels (1, 2, or 3%) increased the concentrations of ruminal NH_3_-N due to greater concentrations of CP in these diets. However, the reasons why *C. vulgaris* at 2% level and *M. oleifera* at lower levels up to 40% levels showed lower NH_3_-N are not clear, but it may be due to interaction of *C. vulgaris* with NH_3_-N producing microbiota (Polyorach et al. 2014). *M. oleifera* in the rations containing 1% and 2% *C. vulgaris* microalgae increased the concentrations of ruminal total VFA and propionate, which is an indication of improved diet fermentability as the VFA are the main end products of ruminal carbohydrate fermentation. However, total VFA and propionate concentrations were not affected at 3% *C. vulgaris* level. In an in vivo study with goats, the diet containing *M. oleifera* at 20% and 40% levels and 1% *C. vulgaris* improved total VFA and propionate concentration (Kholif et al. 2022). Ruminal bacteria degrade structural carbohydrates (cellulose and hemicellulose) and produce acetate. Therefore, the increases in acetate concentration could be attributed to increased activity of cellulolytic and hemicellulolytic bacteria (Carro et al. 2009). Abdel-Raheem and Hassan (2021) observed that substituting soybean meal in the diet of calves with *M. oleifera* leaf meal at 50 and 75% decreased the concentration of NH_3_-N, total protozoal abundance, and acetate to propionate ratio in the rumen. The low replacement level (i.e., 50%) increased the concentrations of acetic, propionic, and isobutyric acid and molar proportion of propionic acid compared with the control (without *M. oleifera*) and 75% replacement level. Low level of *C. vulgaris* may provide the fermentation medium with some growth-stimulating substances including S-nucleotide adenosyl peptide, which can improve nutrient digestibility (Yan et al. 2012). The supplementation of *C. vulgaris* increased the abundances of some ruminal bacteria in vivo (Tsiplakou et al. 2017) and in vitro (Fievez et al. 2007). Tsiplakou et al. (2017) observed that a diet supplemented with *C. vulgaris* changed ruminal cellulolytic and proteolytic bacterial populations and cellulase and protease activity. As previously noted with biogas production, high *C. vulgaris* levels (i.e., 2 and 3% *C. vulgaris*), however, negatively affected fermentation and degradability compared to the low level (i.e., 1% *C. vulgaris*). Some microalgae are reported to contain antimicrobial activity due to the presence of alkaloids, exopolysaccharides, fatty acids, and cyclic peptides (Abedin and Taha 2008). Also, *C. vulgaris* contains phenolic substances, unique polysaccharides, and aromatic compounds, which had a nutritional and ecological importance to the animals fed diets containing *C. vulgaris* (Kholif and Olafadehan 2021b).

## Conclusions

*M. oleifera* silage can replace the concentrate feed mixture up to 30% with positive effects on ruminal fermentation, gas production, and degradability with inhibition of CH_4_ production. *C. vulgaris* at 1% along with *M. oleifera* silage up to 30% in the diets showed additive effects on ruminal fermentation and CH_4_ inhibition. However, *C. vulgaris* at 2 or 3% level exerted negative effects on ruminal fermentation and nutrient degradability though higher levels exerted stronger CH_4_ reducing effect. Although there was no interaction between *M. oleifera* and *C. vulgaris* on most measured ruminal fermentation, a few important variables such as concentrations of total VFA, propionate, and NH_3_-N were affected by the interaction effect. This indicates that there is need a synergy between these two factors in enhancing overall ruminal fermentation. Further in vitro and in vivo studies are required to investigate different levels of *M. oleifera* silage in the presence of different levels of *C. vulgaris* microalgae on the production performance, nutrient utilization, CH_4_ production, ruminal microbiota modulation, and health of ruminants at different stages of production.

## Data Availability

The datasets used and/or analyzed during the current study are available from the corresponding author on reasonable request.
